# A Survey of Otolaryngology Residency Programs on Adapting to the United States Medical Licensing Examination (USMLE) Step 1 Transitioning to Pass/Fail

**DOI:** 10.7759/cureus.45227

**Published:** 2023-09-14

**Authors:** Lydia C Yang, Andrew Bonner, Om U Patel, William Haynes, Kaitlin Burge, Ishant Yadav, Nicholas J Van Wagoner, Benjamin J Greene, Jessica W Grayson

**Affiliations:** 1 Otolaryngology, Head and Neck Surgery, University of Alabama at Birmingham Heersink School of Medicine, Birmingham, USA; 2 Medicine, University of Alabama at Birmingham Heersink School of Medicine, Birmingham, USA

**Keywords:** clinical knowledge, united states medical licensing examination (usmle), medical student exams, otolaryngology residency, usmle step 2 ck, step 1 change, pass/fail usmle step 1

## Abstract

Objectives

In February 2020, the National Board of Medical Examiners (NBME) announced that the United States Medical Licensing Examination (USMLE) Step 1 licensing examination would change from a numerical score to Pass/Fail (P/F). After implementation, many believe that USMLE-Step 2-Clinical Knowledge (CK) will become an important metric for students applying to otolaryngology (ENT). The purpose of this study is to determine factors important to resident selection after these changes.

Methods

A survey containing 15 questions related to resident selection practices and how changing USMLE Step 1 to P/F would impact future resident selection was designed. It was distributed to all ENT residency programs accredited by the Accreditation Council for Graduate Medical Education (ACGME).

Results

Forty percent of programs responded; 66% (95% confidence interval (CI): 51.1%-78.4%) felt that changing Step 1 scoring would not lead to students being more prepared for clinical rotations; 55% believe class rank will increase in significance (95% CI: 35.7%-64.3%). There was also an increase in the importance of Step 2 CK, which had a mean ranking of 10.67 prior to changes in Step 1 scoring and increased to 7.80 after P/F.

Conclusions

The changes in Step 1 scoring will likely lead to increasing importance of other objective measures like class rank or Step 2 CK. This may defeat the intended purpose put forth by the NBME. Therefore, further guidance on measures correlated with student performance as a resident will be integral to the selection process.

## Introduction

Otolaryngology (ENT) residency positions have traditionally been competitive positions to attain based on data from the National Resident Matching Program (NRMP) [1]. In 2022, the total number of applicants for ENT residency positions was 556, and the total number of positions offered was 361 [2]. Based on recent data (2021) [1], 28.0% of US applicants to ENT residency programs went unmatched, which was the highest rate of unmatched applicants compared to all other specialties for that year. Given the highly competitive nature of ENT residency positions, objective measures of scholastic achievement have been integral for residency program directors to decide between highly competitive candidates. One of the primary tools used to assess applicants to ENT programs has been the United States Medical Licensing Examination (USMLE) Step 1, a standardized exam typically taken by U.S. medical students upon completion of pre-clinical coursework. In 2022, the mean Step 1 score of applicants who applied to ENT residency programs and did not match was 243, while the average Step 1 score for applicants across all specialties who matched with their specialty of interest was 236 [2]. Beginning January 26, 2022, the USMLE began reporting scores as pass/fail (P/F) instead of the traditional three-digit numeric scoring system [3].

Even before the change in scoring was announced, many within the medical community outlined problems that were created by relying so heavily on the exam for evaluation purposes. Some made the case that Step 1 scores were never intended or verified as a way to determine substantial differences in medical knowledge between two applicants with small differences in scores. Rather, the test was initially developed to determine whether medical students would be eligible for state licensure at a later stage in training [4]. Student well-being and long-term burnout from stress surrounding Step 1 preparation have also been referenced as reasons for change [5-6]. Other analyses indicated that socioeconomic factors may also play a factor in performance on the Step 1 exam, with the assumption that more affluent students or those with more resources may be able to access more study materials or attend medical schools that provide more instructional material and support than the less affluent students [4,7].

Currently, it is unclear how the change in Step 1 scoring will affect applicants to ENT residency programs. Based on an initial review of literature focused on this subject matter, opinions range widely regarding the exact effects of this change, but most agree that different objective measures of applicant preparedness will replace Step 1 in the level of importance during admissions decisions [8]. The goal of this study is to assess how ENT program directors and assistant program directors believe changes in the Step 1 scoring system will impact resident selection. This article was previously presented as a meeting abstract at the Combined Otolaryngology Spring Meetings (Triological Society) on May 4, 2023.

## Materials and methods

In order to assess how changes in Step 1 scoring will affect the evaluation of medical students applying to ENT residency programs, a Qualtrics survey was created by medical students and physicians at our institution and sent to 125 Accreditation Council for Graduate Medical Education (ACGME)-accredited ENT residency programs across the country. Contact information for residency programs was obtained using the American Medical Association's (AMA) FREIDA^TM^ database. Results were obtained between June 2021 and July 2022. The present study is part of a larger study [9] that sent surveys to residency programs in 25 different specialties. Institutional Review Board (IRB) approval from the University of Alabama at Birmingham was obtained (approval number: IRB-300007220) prior to the study.

Survey components

Questions and topics asked in the survey were developed based on factors included in annual reports from the National Resident Matching Program (NRMP) [1-2]. The survey contained three primary components. The first component asked about program demographics, such as whether the institution was a Top 15 National Institutes of Health (NIH)-funded program and whether it was an academic, academic-affiliated, or community program.

The second component asked programs seven questions about their opinion on using Step 1 and Step 2 to predict a resident’s ability to perform clinically and on specialty board exams. It also asked if medical schools should report other objective factors, such as shelf exam scores and student class ranking, after the change to P/F. Responders could choose from three different answer options: "yes", "neutral", or "no".

The third component had respondents rank 17 applicant factors in order from most important to least important when evaluating applicants for residency. These factors included the following (in no particular order): Step 1 score, mean number of research experiences in ENT, number of abstracts, presentations, and publications, membership in Gold Humanism Honor Society, volunteering experiences, Alpha Omega Alpha membership, clerkship grades, Dean’s letter, personal statement, preclinical grades, letters of recommendation from ENT specialists, class rank/quartile, completion of an away rotation at an ENT program at another institution, completion of a graduate degree (e.g., Master of Public Health (MPH), PhD, etc.), leadership experiences, Step 2 clinical knowledge (CK) score, and graduation from one of the top 40 NIH-funded schools. The following question removed the Step 1 score from the list of applicant factors and asked programs to rank the remaining 16 criteria in order of importance. This reflected the change in Step 1 scoring to P/F. A copy of the survey can be found in the appendices (Appendix A).

Analysis

The results from the survey questions were analyzed in RStudio (RStudio Team, 2020, Boston, MA, USA) using chi-squared tests with 95% confidence intervals. Questions related to ranking important resident factors were analyzed with IBM SPSS Statistics software for Macintosh, Version 29.0 (IBM Corp., Armonk, NY, USA), using a Wilcoxon rank sum test with a significance level of p < 0.05.

## Results

**Figure 1:**
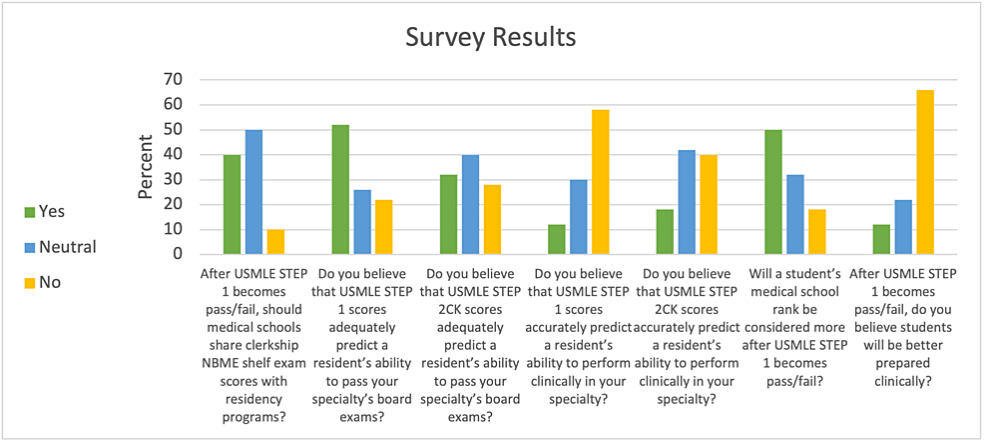
Results for six survey questions USMLE: United States Medical Licensing Examination; NBME: National Board of Medical Examiners; CK: clinical knowledge

Fifty residency programs (40%) responded to the survey. Twenty-six (52%) of respondents believed Step 1 scores were able to predict a resident’s ability to pass ENT board exams, but only 16 respondents (32%) found this to be true for Step 2 CK. Thirty-three (66%) did not believe that students would be better prepared clinically after the change to P/F. Half of the respondents (50%) said medical school ranking would be considered more after P/F, and 20 programs (40%) thought schools should share NBME shelf exam scores with programs. Survey results are displayed in Figure 1.

Thirty programs (24%) responded to the ranking questions. For ranking criteria, programs listed letters of recommendation, Step 1 scores, and the mean number of ENT abstracts, presentations, and publications as the three most important factors before the change to P/F. After P/F, the mean number of research experiences was ranked third, but ENT letters of recommendation and the mean number of ENT abstracts, presentations, and publications remained in the top three. The same three criteria (preclinical grades, graduate degree, and graduating from one of the 40 U.S. medical schools with the highest NIH funding) remained the least important. Notably, Step 2 CK had the largest change in mean ranking, going from 10.67 to 7.80. Full ranking results are listed in Table 1.

**Table 1:** Average ranking criteria before (left) and after (right) Step 1 P/F.  The text in bold designates a significant change in criteria ranking (p < 0.05). Criteria marked with an asterisk (*) were tied. P/F: pass/fail; CK: clinical knowledge; NIH: National Institutes of Health

Before Step 1 P/F	After Step 1 P/F
Letter of Recommendations in Specialty (2.50 ± 2.11)	Letter of Recommendations in Specialty (2.50 ± 2.15)
Step 1 (5.80 ± 4.90)	Mean Number of ENT Abstracts, Presentations, and Publications (5.30 ± 2.32)
Mean Number of ENT Abstracts, Presentations, and Publications (5.87 ± 2.68)	Mean Number of ENT Research Experiences (5.67 ±3.47)
Clerkship Grades (5.93 ± 3.35)	Clerkship Grades (6.20 ± 3.58)
Mean Number of ENT Research Experiences (6.57 ±3.95)	Personal Statement (6.67 ± 3.74)
Personal Statement (7.03 ± 3.90)	Class Rank/Quartile (7.70 ± 4.79)
Involvement/Leadership (8.53 ± 4.37)	Step 2 CK (7.80 ± 5.03)
Class Rank/Quartile (8.63 ± 4.67)	Alpha Omega Alpha (8.23 ± 3.80)
Dean’s Letter (9.23 ± 4.00)	Involvement/Leadership (8.53 ± 4.27)
Alpha Omega Alpha (9.30 ± 3.97)	Dean’s Letter (8.63 ± 3.45)
Number of Volunteer Experiences (9.83 ± 4.04)	Number of Volunteer Experiences (8.90 ± 3.76)
Away Rotation (10.67 ± 4.49)*	Gold Humanism Honors Society (10.27 ± 3.34)
Step 2 CK (10.67 ± 4.63)*	Away Rotation (10.30 ± 4.03)
Gold Humanism Honors Society (11.23 ± 3.19)	Preclinical Grades (11.27 ± 3.14)
Preclinical Grades (11.73 ± 3.58)	Graduate Degree (13.17 ± 2.97)
Graduate Degree (13.93 ± 3.34)	Graduated From One of the 40 U.S. Medical Schools with the Highest NIH Funding (14.87 ± 2.11)
Graduated From one of the 40 U.S. Medical Schools with the Highest NIH Funding (15.53 ± 2.49)	

## Discussion

According to our study, ENT residency programs have traditionally placed a large emphasis on Step 1 when assessing an applicant’s competitiveness for residency. Survey results indicate that many ENT residency program directors believe a valuable data point to assess an applicant’s preparedness for the clinical and academic demands of residency may have been lost. Our results show that more respondents found Step 1 to be a better predictor than Step 2 CK of whether residents could pass specialty board exams. However, after the change to P/F, it appears that residencies will place a significantly larger weight on Step 2 CK when evaluating applicants.

The goal of transitioning to P/F was to reduce the student stress and burnout that surround an examination. However, our results showing that Step 2 CK will become more important imply that the stress around Step 1 may dissipate but will ultimately be shifted to Step 2 CK. Given that ENT is one of the most difficult specialties for students to match, it will be hard for students to determine whether they are competitive applicants when an important component of their application will not be completed until the end of their third year of medical school. By this time, most students have chosen a specialty and are tailoring their final year of medical school to their field of choice.

Despite the removal of Step 1 numerical scores for applicants, our results show that program directors continue to value ENT letters of recommendation as the most important when evaluating residency applicants. The number of research publications and experiences also remain important factors. Students wanting to apply to ENT should continue to consider these criteria when preparing for residency with a pass/fail Step 1 score. However, not every medical school has an ENT department or the chance to explore the field during clinical rotations, and these barriers remain for students.

Other studies surveying the impact of Step 1 scoring transitioning to P/F for ENT residency programs have shown similar results. Many survey respondents do not support the transition to P/F and do not think it will improve students’ well-being [10-11]. One study examined the correlation between Step 1 and other objective applicant factors using two cycles of ENT applications received by their academic institution [12]. Step 2 CK had a moderate correlation with Step 1 scores, but other metrics such as the number of publications and presentations were found to have no significance [12]. This further supports our findings that by moving to P/F, Step 1 will displace, not alleviate, the stress associated with performing well on a single test.

In addition to the current literature, our results revealed that respondents did not think Step 1 was an accurate indicator of whether a resident would perform well clinically in ENT. However, programs did find it better than Step 2 CK for predicting an applicant’s ability to pass ENT board exams. Furthermore, programs did not believe applicants would be better prepared clinically. This raises concerns as to whether the transition to P/F may actually hurt students during clinical rotations and even during residency training. It suggests that keeping numerical scores for both exams may be beneficial as they predict different things: the ability to perform well on board exams for Step 1 and clinical skills for Step 2. The two exams are not interchangeable, and each contains its own unique objectives.

Our study has several limitations, one of which is our response rate of 40%. We acknowledge it is difficult to draw major conclusions from a limited sample, but we believe this can still offer students guidance during the coming application cycle when the majority of applicants will have P/F Step 1 scores. Furthermore, data were collected over the course of a year, and it is possible that programs are still actively determining what they will ultimately value when looking at future applications. Future studies after the 2024 NRMP application cycle will be able to further elucidate what programs look for in ENT applicants. We also acknowledge that this study focuses on ENT programs specifically and that the transition to P/F impacts each specialty differently [13-15].

## Conclusions

Based on survey results, once Step 1 becomes P/F, Step 2 CK scores will become more important in ranking potential resident applicants. However, ENT letters of recommendation remain the most important factor when evaluating applicants. Most ENT programs do not believe the transition to P/F will better prepare students for residency. Further guidance on measures correlated with student performance as a resident will be important.

## Figures and Tables

**Figure 2:**
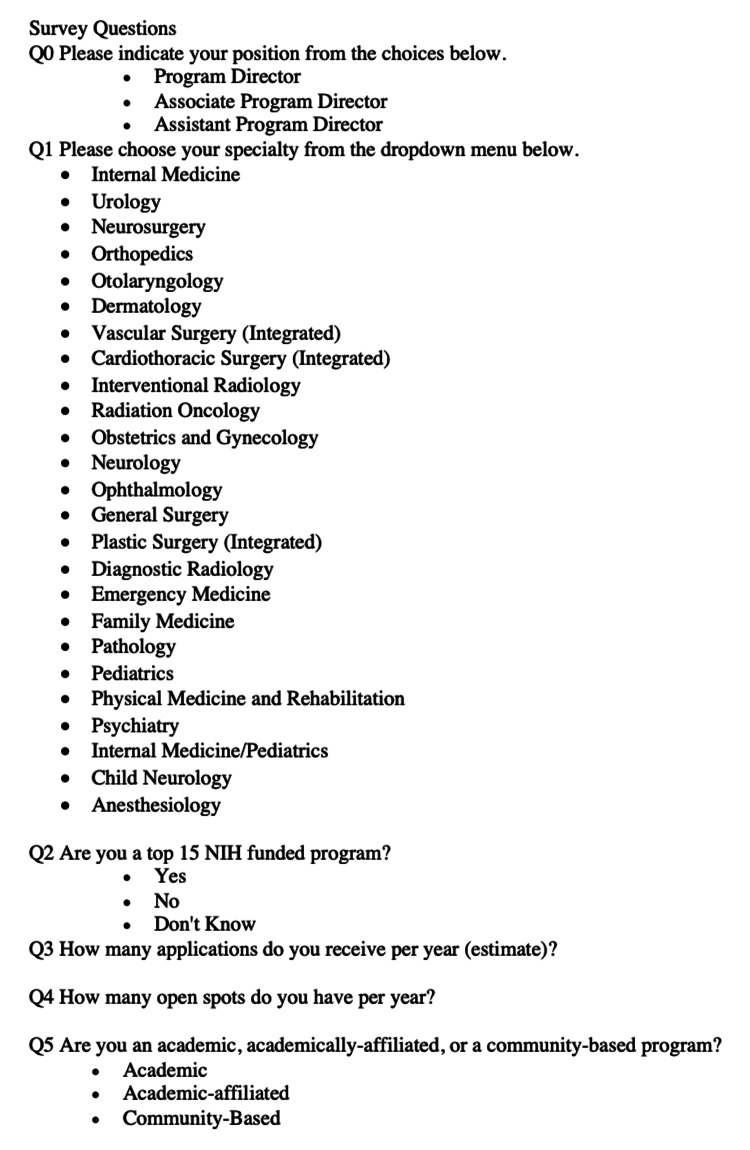
Page 1 of the survey questions

**Figure 3:**
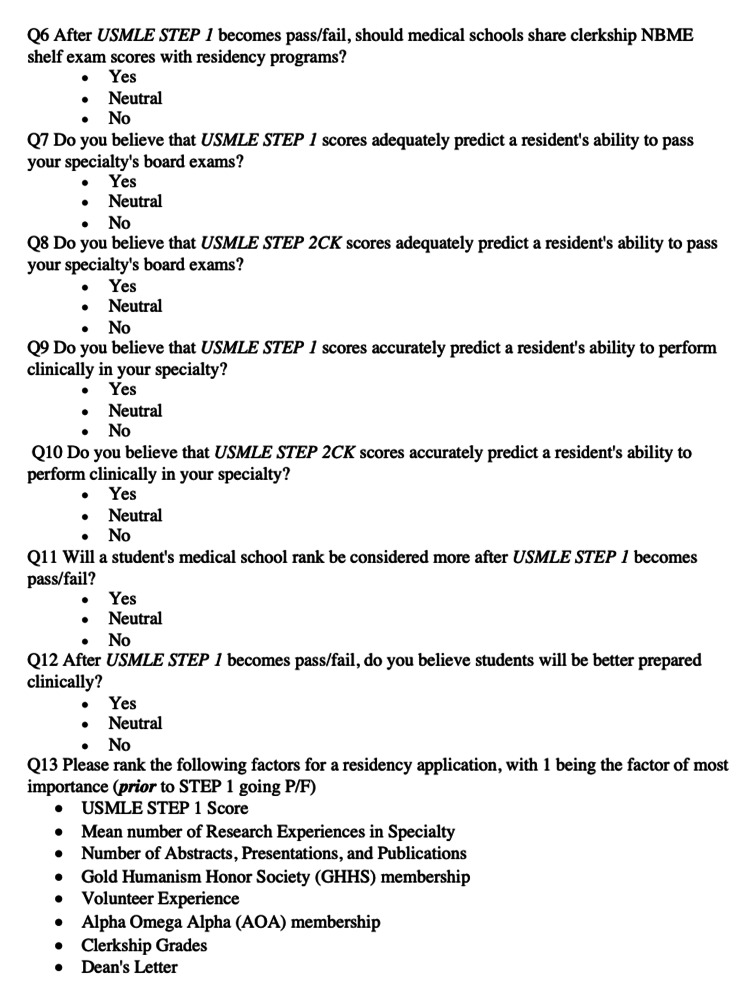
Page 2 of the survey questions

**Figure 4:**
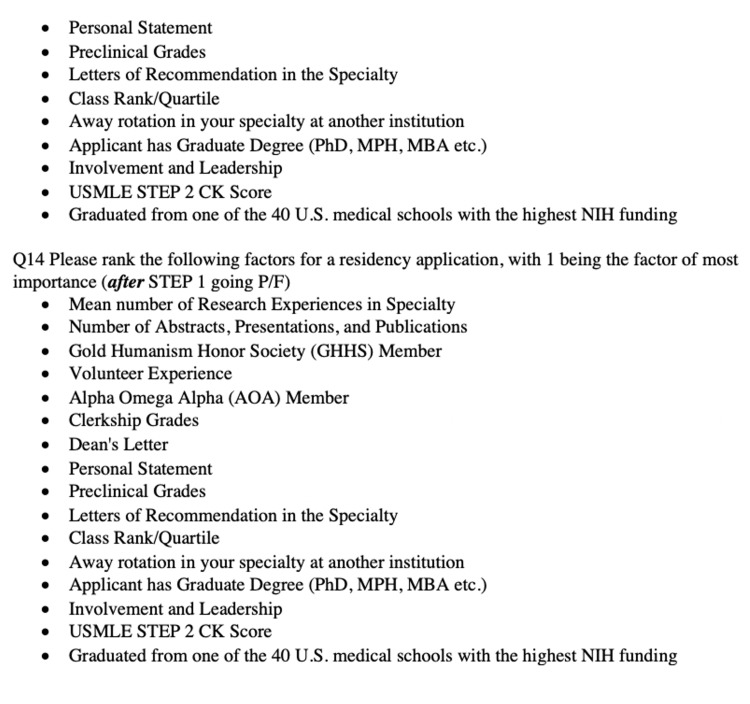
Page 3 of the survey questions

## Appendices

Appendix A 
